# Receptor Activity-modifying Proteins 2 and 3 Generate Adrenomedullin Receptor Subtypes with Distinct Molecular Properties*[Fn FN2]

**DOI:** 10.1074/jbc.M115.688218

**Published:** 2016-03-24

**Authors:** Harriet A. Watkins, Madhuri Chakravarthy, Rekhati S. Abhayawardana, Joseph J. Gingell, Michael Garelja, Meenakshi Pardamwar, James M. W. R. McElhinney, Alex Lathbridge, Arran Constantine, Paul W. R. Harris, Tsz-Ying Yuen, Margaret A. Brimble, James Barwell, David R. Poyner, Michael J. Woolley, Alex C. Conner, Augen A. Pioszak, Christopher A. Reynolds, Debbie L. Hay

**Affiliations:** From the ‡ School of Biological Sciences,; the § Maurice Wilkins Centre for Molecular Biodiscovery, and; the ‖ School of Chemical Sciences, University of Auckland, Auckland 1010, New Zealand,; the ¶ School of Biological Sciences, University of Essex, Wivenhoe Park, Colchester CO4 3SQ, United Kingdom,; the ** School of Life and Health Sciences, Aston University, Aston Triangle, Birmingham B4 7ET, United Kingdom,; the ‡‡ School of Clinical and Experimental Medicine, University of Birmingham, Edgbaston, Birmingham B15 2TT, United Kingdom,; the §§ Department of Biochemistry and Molecular Biology, University of Oklahoma Health Sciences Center, Oklahoma City, Oklahoma 73104

**Keywords:** allosteric regulation, cardiovascular disease, conformational change, G protein-coupled receptor (GPCR), molecular modeling, GPCR, RAMP, adrenomedullin, extracellular loops, receptor activity-modifying protein

## Abstract

Adrenomedullin (AM) is a peptide hormone with numerous effects in the vascular systems. AM signals through the AM_1_ and AM_2_ receptors formed by the obligate heterodimerization of a G protein-coupled receptor, the calcitonin receptor-like receptor (CLR), and receptor activity-modifying proteins 2 and 3 (RAMP2 and RAMP3), respectively. These different CLR-RAMP interactions yield discrete receptor pharmacology and physiological effects. The effective design of therapeutics that target the individual AM receptors is dependent on understanding the molecular details of the effects of RAMPs on CLR. To understand the role of RAMP2 and -3 on the activation and conformation of the CLR subunit of AM receptors, we mutated 68 individual amino acids in the juxtamembrane region of CLR, a key region for activation of AM receptors, and determined the effects on cAMP signaling. Sixteen CLR mutations had differential effects between the AM_1_ and AM_2_ receptors. Accompanying this, independent molecular modeling of the full-length AM-bound AM_1_ and AM_2_ receptors predicted differences in the binding pocket and differences in the electrostatic potential of the two AM receptors. Druggability analysis indicated unique features that could be used to develop selective small molecule ligands for each receptor. The interaction of RAMP2 or RAMP3 with CLR induces conformational variation in the juxtamembrane region, yielding distinct binding pockets, probably via an allosteric mechanism. These subtype-specific differences have implications for the design of therapeutics aimed at specific AM receptors and for understanding the mechanisms by which accessory proteins affect G protein-coupled receptor function.

## Introduction

The endothelium-derived peptide hormone adrenomedullin (AM)^6^ is a protective factor in the cardiovascular system and a biomarker for cardiovascular disease (1–5). AM administration in human subjects has several positive outcomes, significantly improving patient recovery from myocardial infarction, inhibiting myocyte apoptosis, reducing mean pulmonary arterial pressure, and increasing cardiac output in heart failure patients (1, 5–7). However, serious adverse hypotension in some patients, coupled with rapid metabolism of the peptide, means that optimal targeting of the AM system still needs to be achieved (7, 8). The pro-angiogenic effects of AM mean that receptor agonists or antagonists could be useful in a range of other conditions, such as lymphedema or cancer (9). Realizing any of these therapeutic goals, however, requires a much greater understanding of AM receptor biology. Here we explore receptor architecture to lay the foundations for the design of selective AM receptor ligands.

AM signals through two receptors. These both contain the calcitonin receptor-like receptor (CLR), a class B G protein-coupled receptor (GPCR) that has an absolute requirement for association with a receptor activity-modifying protein (RAMP) for ligand binding and receptor activation to occur. Association of CLR with RAMP2 generates the AM_1_ receptor, whereas CLR with RAMP3 forms the AM_2_ receptor (10).

The AM_1_ receptor has an important role in cardiovascular system development. Deletion of the genes for AM, CLR, or RAMP2 results in embryonic lethality due to the development of hydrops fetalis and cardiovascular abnormalities (11–13). For example, *Adm*^−/−^ mice have small and disorganized hearts (13). Cardiomyocyte-specific RAMP2 knock-out disrupted cardiac metabolism and homeostasis by causing cardiac dilation and changes in mitochondrial structure (14). Furthermore, targeted RAMP2 overexpression in vascular smooth muscle suggests that the AM_1_ receptor could protect against vascular remodeling invoked by prolonged hypertension (15).

RAMP3 knock-out mice give important insight into the likely role of the AM_2_ receptor in cardiac biology. Unlike RAMP2 knock-out mice, these animals survive into old age and exhibit normal angiogenesis (12, 16). When challenged by crossing *Ramp3*^−/−^ with *RenTgMK* mice (a genetic model of angiotensin II-mediated cardiovascular disease), sex-dependent cardiovascular phenotypic differences emerge (*i.e.* renal failure and cardiac hypertrophy occur only in male mice) (16). A separate *Ramp3*^−/−^ model exhibited narrowed lymphatic vessels, impaired lymphatic drainage, and thus post-operative lymphedema and prolonged inflammation (17).

Thus, the AM_1_ and AM_2_ receptors have distinct roles. In animal models of cardiovascular disease, both the relative and absolute expression of the AM_1_ and AM_2_ receptor subunits change in different disease states. In the kidney of hypertensive rats, RAMP2 expression decreases, and RAMP3 expression increases (18). Each AM receptor is a potential drug target, and it is important to develop selective molecules for each receptor that can tease out the most beneficial receptor activity. For example, AM_1_ receptor antagonists could be useful anti-angiogenic agents in cancer (9). In cardiovascular disease, either receptor could be a drug target. Receptor-selective molecules are urgently needed to tease out the role of each receptor and enable drug development efforts.

The AM receptors are compelling targets from a drug discovery perspective because of their biological effects and because they belong to the large GPCR superfamily of transmembrane proteins that are the cellular targets for 36% of all approved therapeutics (19). Peptide-binding class B GPCRs (including CLR) maintain the conserved heptahelical conformation observed across the wider superfamily with attendant intracellular loops, extracellular loops (ECLs), and a large extracellular domain (ECD) (20). Class B GPCR peptide ligands are known to interact with the ECD through their C terminus, with a second interaction of their N terminus with the juxtamembrane domain (the ECLs and the upper region of the transmembrane (TM) helices) that initiates receptor activation. However, the fact that the two AM receptors share a common GPCR (CLR) and the natural ligand (AM) makes minimal direct contact with the RAMP ECD (21) makes the design of receptor-specific drugs a challenge. Rational design of specific ligands would therefore benefit from improved knowledge of the full impact of RAMPs upon AM_1_ and AM_2_ receptor structure and function. In the pursuit of AM receptor agonists, a focus on the regions of CLR that trigger signaling is critical.

Here we explore how RAMPs affect the CLR juxtamembrane domain through extensive site-directed mutagenesis and molecular modeling. Our data suggest that RAMP2 and RAMP3 each create unique CLR conformations that may be exploitable for the development of small molecule ligands.

## Experimental Procedures

### 

#### 

##### Materials

Human AM (AM(1–52)) was purchased from American Peptide (Sunnyvale, CA). Forskolin was from Tocris Bioscience (Wiltshire, UK). ALPHAscreen cAMP assay kits were from PerkinElmer Life Sciences. Poly-d-lysine-coated plates were from BD (Auckland, New Zealand). ^125^I-AM(13–52) was from PerkinElmer Life Sciences.

##### Expression Constructs and Mutagenesis

Wild type (WT) human CLR with an N-terminal hemagglutinin (HA) epitope tag, human RAMP2 with an N-terminal FLAG epitope tag, and untagged human RAMP3 were used in this study (22, 23). The HA-CLR mutants and RAMP constructs have been described previously (24–26).

##### Cell Culture and Transfection

Culture of HEK293S cells was performed as described previously (23). Cells were counted using a Countess Counter^TM^ (Invitrogen) and seeded at a density of 15,000 cells/well into 96-well poly-d-lysine-coated plates. For binding assays, 24-well plates were used (22). These were transiently transfected using polyethyleneimine as described previously (27).

##### Synthesis of Alanine-substituted AM(15–52) and Experiments with Phe^18^ AM

For experiments investigating the role of Phe^18^ in the AM peptide, we used an F18A AM(15–52) peptide, alongside a WT AM(15–52) control. As is evident from the data for full-length AM(1–52) and AM(15–52) (Tables 2 and 4), these peptides have equivalent function. The AM(15–52) peptides were synthesized by solid phase peptide synthesis using the Fmoc/*tert*-butyl method on a 0.1-mmol scale. Briefly, Rink amide aminomethyl resin was prepared (28), and the peptide was elongated using a CEM Liberty microwave peptide synthesizer (CEM Corp., Matthews, NC) using 5% (w/v) piperazine containing 0.1 m 6-chlorobenzatriazole in *N*,*N*-dimethylformamide as Fmoc deblocking reagent and *O*-(6-chlorobenzotriazol-1-yl)-*N*,*N*,*N*′,*N*′-tetramethyluronium hexafluorophosphate, and *N*,*N*-diisopropylethylamine as coupling reagents using microwave settings as described previously (29). The peptides were cleaved from the resin with concomitant removal of side chain protecting groups with 94.0% trifluoroacetic acid, 1.0% triisopropylsilane, 2.5% water, and 2.5% 2,2′-(ethylenedioxy)diethanethiol (v/v/v/v) for 2–3 h, precipitated with cold diethyl ether, recovered by centrifugation, dissolved in 50% aqueous acetonitrile containing 0.1% trifluoroacetic acid, and lyophilized. The crude peptides were dissolved in 0.1 m Tris (pH 8.1) at a concentration of 1 mg/ml, and the oxidation (disulfide formation) was allowed to proceed at room temperature open to air. Monitoring by reverse phase HPLC and/or LC-MS indicated that the reaction was typically complete within 1 day. The solution was acidified to pH 2 with 5 m HCl, purified directly by semipreparative reverse phase HPLC using a C18 Gemini (Phenomenex, Torrance, CA) column (10 × 250 mm) at a flow rate of 5 ml/min, and eluted using an appropriate gradient based on the analytical HPLC profile. Fractions containing the pure peptide were identified by electrospray mass spectrometry and/or HPLC, pooled, and lyophilized. All peptides were >95% pure as judged by integration of the HPLC chromatogram at 210 nm, and peptide masses were confirmed by electrospray mass spectrometry.

##### cAMP Assays

We selected the mutants to study based on the boundaries of the ECLs according to our homology model of the calcitonin gene-related peptide (CGRP) receptor (CLR/RAMP1) (26, 30). CLR is predominantly G_s_-coupled, so we characterized AM-stimulated cAMP signaling of alanine (or leucine, where natively alanine) mutants of CLR complexed with either RAMP2 or RAMP3. cAMP assays were performed as described previously using 1 mm isobutylmethylxanthine and a 15-min cell stimulation period (31). cAMP was then quantified using ALPHAscreen on a JANUS automated work station (PerkinElmer Life Sciences).

##### Analysis of Cell Surface Expression of Mutants by ELISA

CLR, RAMP2, and RAMP3 are inefficiently expressed on their own at the cell surface (32). However, when CLR is expressed with either RAMP, a functional AM_1_ or AM_2_ receptor is translocated to the cell surface. We determined expression levels of WT CLR/RAMP2 and CLR/RAMP3 heterodimers and cell surface expression of the mutant receptors as described previously, by measuring HA-CLR (33, 34). Due to the RAMP-dependent effects observed, we first ensured that each RAMP was capable of producing equivalent HA-CLR translocation to the cell surface: HA-CLR cell surface expression with (*A*_490_ − *A*_650/595_) untagged RAMP1, 4.32 ± 0.31 (*n* = 3); Myc-RAMP1, 4.16 ± 0.22 (*n* = 3); untagged RAMP2, 2.81 ± 0.42 (*n* = 3); FLAG-RAMP2, 3.08 ± 0.38 (*n* = 3); or untagged RAMP3, 2.96 ± 0.36 (*n* = 3) (no significant differences by one-way analysis of variance). Thus, RAMP-specific effects of CLR mutations are unlikely to be due to an alteration in receptor density at the cell surface.

##### Radioligand Binding

AM binding assays were performed as described previously, displacing ^125^I-AM(13–52) with unlabeled AM (22).

##### Data Analysis

All experiments were independently replicated at least three times, with two or three technical replicates in each experiment. Data analysis for cAMP assays was performed in GraphPad Prism version 6 (GraphPad Software, La Jolla, CA). Concentration-response curves were initially fitted to a four-parameter logistic equation; in all cases, the Hill slope was not significantly different from unity. Consequently, this was constrained to equal 1, the data were refitted to a three-parameter logistic equation, and pEC_50_ values were obtained. In order to determine *E*_max_ values for the mutant receptor curves, the data were normalized with respect to the fitted minimum and maximum of the WT curve. The combined normalized data sets were generated by combining the mean of the data points from the curves of each individual experiment. Variations in pEC_50_ between WT and mutant receptors were analyzed for statistical significance using an unpaired *t* test on the values obtained before curve normalization (*, *p* < 0.05; **, *p* < 0.01; ***, *p* < 0.001). *E*_max_ values expressed as a percentage of WT were analyzed similarly. A Δlog pEC_50_ of ≥0.5 and a ≥30% *E*_max_ difference (compared with WT) coupled to significance at the *p* < 0.05 level were used to identify residues with an unambiguous effect.

To further identify mutants that discriminated between AM_1_ and AM_2_ receptors, the differences in relative activity (RA) between the WT and mutant receptors were considered (35). The log(RA) for each mutant and corresponding WT were calculated as log(mutant *E*_max_/mutant EC_50_) and log(WT *E*_max_/WT EC_50_). The mutant value was subtracted from the WT value to obtain Δlog(RA). Δlog(RA) values different from 0 were identified using multiple *t* tests with the false discovery rate set at 1%; differences between Δlog(RA)at the AM_1_ receptor and AM_2_ receptor were investigated by a two-way analysis of variance followed by Sidak's multiple comparison test to compare individual means. Radioligand binding was analyzed in GraphPad Prism version 6 to a three-parameter logistic equation to obtain the pIC_50_ and maximum specific binding.

For ELISA, values were normalized to WT HA-CLR/RAMP as 100% and empty vector-transfected cells as 0%. Statistical significance between WT and mutants was determined using the 95% confidence interval.

##### AM Peptide Structure Model

**FIGURE 1.**
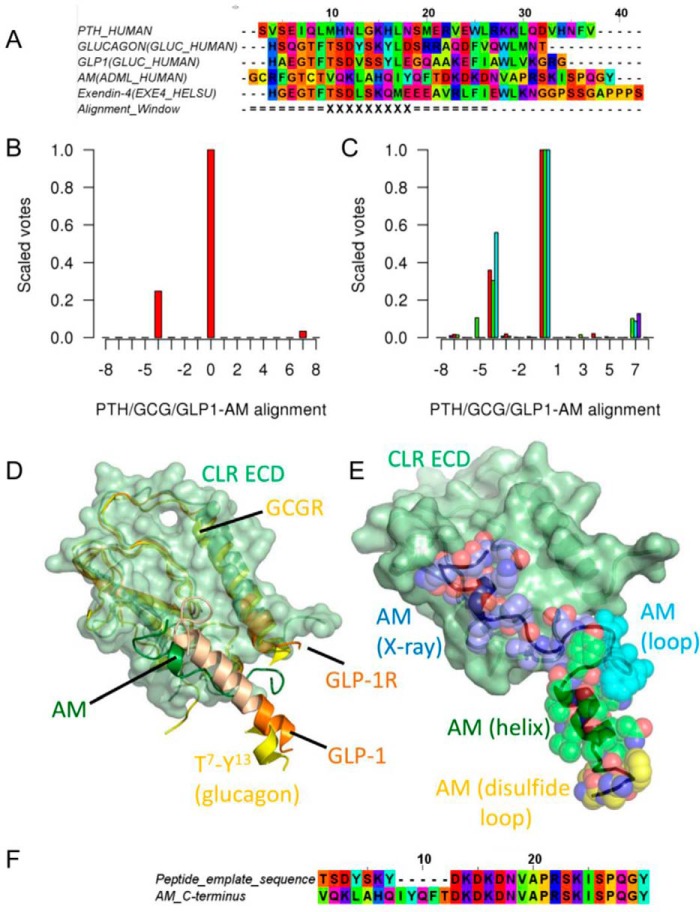
**Modeling the AM peptide.** *A*, selected class B peptide alignments. Homologs of each of PTH, glucagon, and GLP-1 were aligned against AM homologs in a multireference profile alignment, as described by Lock *et al.* (36), over the helical region denoted *X. B*, the multireference alignment scores. Alignment 0, corresponding to the alignment in *A*, has the highest score; the next highest score (alignment −4) corresponds to moving the AM helix 4 residues to the left, but this alternative score is low. *C*, as for *B* but missing PTH (*red*), glucagon (*green*), or GLP-1 (cyan); the results are presented as a control. *D*, a structural alignment of CLR (*light green surface*, *schematic*)/AM(35–52) (*dark green schematic*), GLP-1R (*orange schematic*)/exendin-4 (*wheat*/*orange schematic*), and GCGR (*yellow schematic*)/glucagon Thr^7^–Tyr^13^ (*yellow*). The AM(23–52) comparative modeling template was taken from AM(35–52) and glucagon Thr^7^–Tyr^13^. The exendin-4 is largely *wheat-colored*, but the region corresponding to Thr^7^–Tyr^13^ of glucagon is *orange. E*, the final AM(16–52) structure (*black schematic*, used as one of the templates for modeling the AM receptor) structurally aligned to the CLR ECD. The various components of AM are shown as *color-coded transparent spheres*: *yellow*, carbon atoms (disulfide-bonded loop); *green*, carbon atoms (helix); *cyan*, carbon atoms (loop); *blue*, carbon atoms (from the original x-ray structure). The final structure is very similar to this initial template structure. *F*, the alignment for the comparative modeling of AM(23–52).

The AM peptide structure (Fig. 1) was modeled from the known structures of its component parts (the disulfide-bonded region, the helical region, and the ECD region). The key stages in this modeling involved (i) the use of an in-house multiple-reference sequence alignment method tailored for aligning helices with low sequence identity (36) and (ii) the comparative modeling capabilities of PLOP (37). There is little structural information for full-length AM in its receptor-bound conformation, making structure-based sequence alignments difficult. Moreover, class B GPCR peptide ligands appear to lie in a number of distinct groups (38), so sequence alignment is not trivial. Consequently, separate alignments of the glucagon, GLP-1, PTH, and AM sequences were generated by ClustalX (39). The helical region of the AM peptide homologs, as indicated by the NMR structure (PDB code 2L7S) (40), was aligned to those of the equivalent helical region in the glucagon/GLP-1/PTH family of peptides using an in-house multiple-reference method tailored for aligning helices with low sequence identity (36) that is a development of the methods of reference (41). The alignment is given in Fig. 1*A*; the alignment scores shown in Fig. 1*B* (and Fig. 1*C*) give strong support for the proposed alignment over the only plausible alternative involving a shift left of the AM helix by 4 positions. The AM/CLR/RAMP2 (PDB code 4RWF) ECD (21), the GLP-1/exendin-4 structure (PDB code 3C59) (42), and the glucagon model structure (43) were structurally aligned using the SALIGN module of MODELER (44) (Fig. 1*D*), from which a template was constructed using Asp^35^–Tyr^52^ from the AM x-ray structure and Thr^7^–Tyr^13^ of the glucagon model peptide structure, which was preferred over the corresponding (Thr^7^)-Asp^9^-Gln^13^ of exendin-4 because the angle was more appropriate for peptide binding to the TM bundle. The missing loop was inserted using the comparative modeling, loop modeling, and minimization capabilities of PLOP (37) based on the alignment in Fig. 1*F*. The N terminus, taken from Woolley *et al.* (26), was added by structural alignment of the common helical domain using VMD (45), again using the alignment in Fig. 1*A*. The resulting peptide structure of AM(15–52) (structurally aligned to the CLR ECD) is shown in Fig. 1*E*.

##### AM_1_ and AM_2_ Receptor Models

**FIGURE 2.**
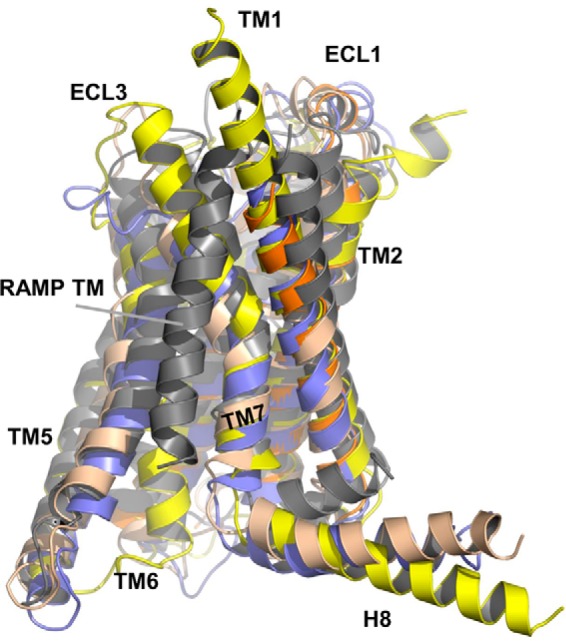
**The template structure of RAMP docked to an active model of CLR.** The template structure (*gray*) was generated as follows. The length of the TM helix for RAMP1 is given as 21 residues by UniProt, but this is too short for a tilted helix to span the membrane. Consequently, for RAMP1, helices of lengths 26, 28, and 30 residues were constructed using Maestro, commencing at Ser^117^, Pro^115^, and Asp^113^, respectively. For RAMP2, helices of length 24, 26, and 28 residues were constructed, commencing at Asp^144^, Pro^142^, and Asp^140^, respectively. For RAMP3, helices of lengths 25, 26, and 28 residues were constructed, commencing at Asp^116^, Pro^115^, and Asp^113^, respectively. The helices were docked using the Cluspro, PatchDock, and SwarmDock servers to two active models of the CLR transmembrane helical bundle (six docking experiments) (30, 65–67); the active explicit membrane CLR model has been shown to be in very good agreement with the x-ray crystal structures of the GCGR and CRF1R (26). Results from each server that were not compatible with the membrane topology were eliminated, and the remaining viable solutions were clustered. Representative solutions were then refined and rescored using the FireDock server (so that poses generated by the different servers are treated equally) (68, 69). The three best poses (on the basis of lowest energy and geometry consensus) were then docked using RosettaDock (70–72). The consensus result showed a preference for the helix to dock to TM7 of the active receptor, in agreement with experimental results that indicate an interaction with TM6/7 (60). The active AM_1_ (*light blue*) and AM_2_ (*wheat*) model TM domains, the inactive GCGR (*yellow*), and TM1–TM4 of inactive CRF1R (*orange*) structures, superimposed over TM1, TM2, TM3, the top of TM4 (because of irregularities in the GCGR x-ray structure; *c.f.* CRF1R), and TM7, are also shown. TM5, TM6, and the top of TM7 were omitted from the fitting because of differences in active and inactive structures in this region. All root mean square deviations were <2 Å.

**FIGURE 3.**
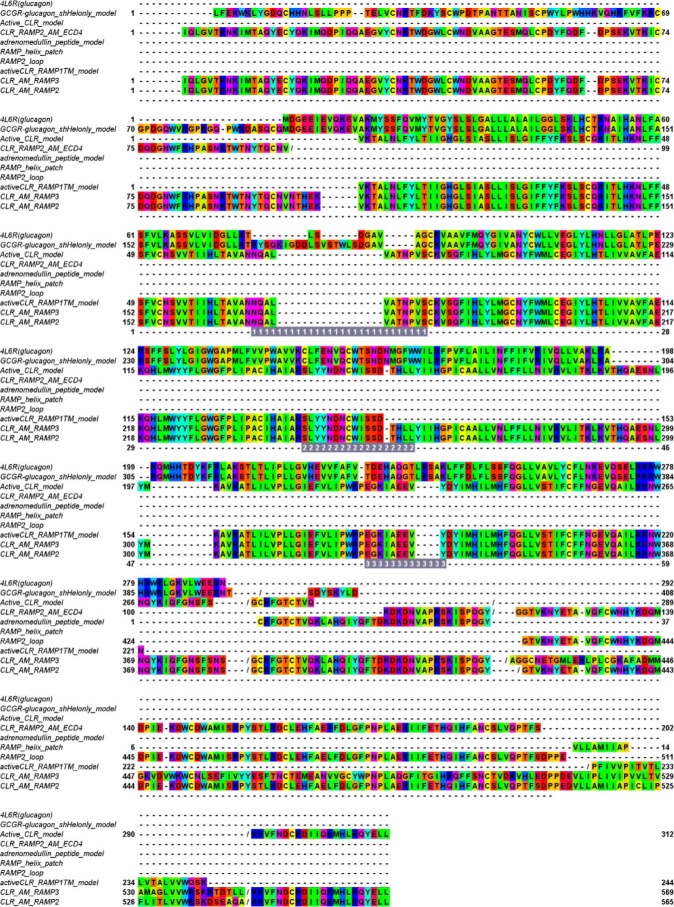
**The alignment for comparative modeling.** The alignment was generated by structural alignment of the templates using the SALIGN module of MODELER and refined using Jalview (73). The residues are *color-coded* according to their properties as follows: *blue*, positive; *red*, negative or small polar; *purple*, polar; *green* large hydrophobic; *yellow*, small hydrophobic; *cyan*, polar, aromatic. This corresponds to the “Taylor” scheme, as implemented in Jalview. The extracellular loops are denoted by *gray shading* and the *loop number*.

Comparative AM_1_ and AM_2_ receptor models were generated using MODELER version 9.12 (44), essentially from two x-ray structures, namely the AM CLR-RAMP2 ECD complex (21) (PDB code 4RWF) and the glucagon receptor (GCGR) TM domain (43) (PDB code 4L6R). The GCGR was preferred over the corticotropin-releasing factor 1 receptor (CRF1R) TM structure because of its overall conformation and compatibility with the full GCGR model (43), but part of the superior quality CRF1R structure (as denoted by ERRAT (46)) was used in subsequent refinement. In addition, model structures for the full GCGR model (43) containing only Ser^8^–Asp^15^ of glucagon (*c.f.* Fig. 1*D*), the full-length AM peptide (Fig. 1*E*), CGRP(1–7) docked to an active model of CLR (26), and a model of the RAMP1 TM helix docked to TM7 were used (Fig. 2). The active character of the model was also imposed by including TM5-6 of an active CLR model derived from the β_2_-adrenergic receptor active complex (47); this template also contained the C-terminal peptide of the G protein, G_s_ (Arg^373^–Leu^394^). Each of these structural templates contained information on part but not all of the desired structure and was linked via a global alignment (Fig. 3). In addition, we also included short N- and C-terminal extensions (6 and 5 residues, respectively) to the RAMP TM helix and the RAMP ECD to prevent the linker between them from becoming entangled in the bulk of the receptor. Within this alignment, the position of the gap in the CLR sequence between the ECD and TM1 relative to the longer human glucagon receptor sequence was determined by analysis of gaps in similar subsets within the glucagon multiple-sequence alignment (48). Two thousand models were generated, and the model having the lowest (best) DOPE score was chosen for further refinement. ECL1 was refined using MODELER from TM1–4 of a CLR model derived from the CRF1R structure in which variability (30, 49, 50) was used to orient the CLR ECL1 helix, as in a recent GLP-1 receptor model (51). The ECLs and the RAMP linker (here defined as the region connecting the extracellular helical domain and the TM helix (*i.e.* residues Val^134^–Leu^147^ for RAMP2 and Val^106^–Leu^119^ for RAMP3)) were refined using PLOP, which has been shown to perform well in GPCR loop modeling (37); this refinement removed any bias introduced by the extensions. The final models were minimized using PLOP (37).

Druggability was assessed using the PockDrug (52, 53) and DoGSiteScorer Web servers (54); pocket hull volumes (which include atoms within the druggable binding pockets) were also determined using PockDrug; distances were measured using the PyMOL Molecular Graphics System (version 1.7.4; Schrödinger, LLC, New York), which was also used for image generation. The models are available as supplemental models 1 and 2.

## Results

### 

#### 

##### Receptor Cell Surface Expression

The cell surface expression levels of the WT AM_1_ and AM_2_ receptors were not significantly different (see “Experimental Procedures”). The cell surface expression of all mutant receptors showed very few significant differences compared with WT (Table 1). L351A and E357A CLR showed an ≥80% reduction of cell surface expression with both RAMPs, suggesting that these mutations caused the receptors to fail quality control processes prior to reaching the cell surface. Further data for these mutants is not discussed.

**TABLE 1:** **Cell surface expression of AM_1_ and AM_2_ receptors expressed as a percentage of the wild type receptor** Data are mean ± S.E. of *n* = 3–4 individual experiments. *95% confidence interval does not include 100%.

	TM2-ECL1-TM3			TM4-ECL2-TM5			TM6-ECL3-TM7	
	AM_1_ receptor	AM_2_ receptor		AM_1_ receptor	AM_2_ receptor		AM_1_ receptor	AM_2_ receptor
	%	%		%	%		%	%
L195A	91.2 ± 2.08	81.4 ± 25.9	A271L	118.8 ± 13.2	118.8 ± 20.6	F349A	98.5 ± 11.3	93.9 ± 22.5
T196A	94.9 ± 17.8	90.5 ± 20.1	I272A	115.9 ± 15.6	64.1 ± 23.5	V350A	104.2 ± 8.06	106.5 ± 33.4
A197L	111.4 ± 7.28	85.9 ± 21.2	A273L	112.3 ± 11.6	107.7 ± 6.74	L351A	9.75 ± 3.17*	21.9 ± 12.3*
V198A	105.8 ± 14.1	84.3 ± 19.0	R274A	98.1 ± 17.3	107.2 ± 14.4	I352A	68.9 ± 10.2*	121.2 ± 13.7
A199L	99.8 ± 7.56	136.7 ± 76.6	S275A	77.3 ± 10.1	113.0 ± 25.3	P353A	94.2 ± 8.95	104.0 ± 23.8
N200A	100.1 ± 6.69	48.4 ± 8.62	L276A	73.8 ± 10.2	110.3 ± 21.7	W354A	85.9 ± 9.30	103.7 ± 24.0
N201A	81.9 ± 15.9	38.9 ± 13.4	Y277A	94.0 ± 7.26	132.4 ± 23.0	R355A	114.7 ± 13.0	86.5 ± 11.8
Q202A	94.8 ± 1.92	83.2 ± 10.2	Y278A	75.8 ± 27.6	154.1 ± 27.1	P356A	101.3 ± 3.59	87.6 ± 13.3
A203L	95.5 ± 9.67	72.1 ± 18.4	N279A	97.0 ± 6.30	96.8 ± 17.0	E357A	17.2 ± 4.85*	15.1 ± 9.97*
L204A	98.3 ± 2.83	114.0 ± 15.8	D280A	155.8 ± 68.9	126.1 ± 16.8	G358A	108.2 ± 7.72	85.7 ± 9.81
V205A	117.3 ± 6.49	88.7 ± 13.1	N281A	108.1 ± 2.90	119.9 ± 2.97	K359A	99.6 ± 2.63	76.3 ± 11.9
A206L	92.9 ± 8.45	91.4 ± 13.1	C282A	64.9 ± 16.8	100.7 ± 11.7	I360A	85.8 ± 3.81	82.5 ± 7.48
T207A	99.9 ± 1.52	82.2 ± 15.5	W283A	107.7 ± 11.9	100.4 ± 13.4	A361L	102.2 ± 3.45	83.1 ± 8.75
N208A	98.6 ± 1.02	111.5 ± 18.4	I284A	67.1 ± 8.80	107.6 ± 26.2	E362A	103.8 ± 6.14	75.4 ± 4.93
P209A	100.3 ± 5.96	118.6 ± 34.5	S285A	86.1 ± 20.2	124.7 ± 31.6	E363A	103.0 ± 2.74	75.6 ± 4.22
V210A	100.4 ± 5.63	88.2 ± 15.0	S286A	93.2 ± 12.9	104.7 ± 15.0	V364A	94.0 ± 2.74	100.5 ± 12.5
S211A	97.5 ± 3.73	95.4 ± 18.7	D287A	77.4 ± 13.4	107.3 ± 7.33	Y365A	92.5 ± 6.21	121.3 ± 24.5
C212A	119.0 ± 25.1	93.6 ± 22.1	I288A	94.8 ± 23.5	90.0 ± 7.14	D366A	107.5 ± 4.33	103.1 ± 19.4
K213A	131.3 ± 33.1	84.3 ± 21.3	H289A	93.1 ± 16.2	95.7 ± 12.9	Y367A	110.0 ± 2.03	106.2 ± 16.4
V214A	87.5 ± 9.71	86.6 ± 17.9	L290A	102.5 ± 2.45	98.6 ± 7.36	I368A	90.8 ± 15.2	118.6 ± 24.7
S215A	86.2 ± 10.7	84.5 ± 17.3	L291A	103.5 ± 7.54	130.8 ± 10.3	M369A	82.8 ± 13.9	108.2 ± 25.1
Q216A	95.7 ± 2.47	124.9 ± 23.7	Y292A	83.5 ± 7.47	176.5 ± 40.6			
F217A	101.8 ± 7.45	99.0 ± 12.0	I293A	99.2 ± 1.44	107.9 ± 12.5			
			I294A	103.2 ± 14.7	102.8 ± 29.8			

##### Functional Analysis of Receptor Mutations

**FIGURE 4.**
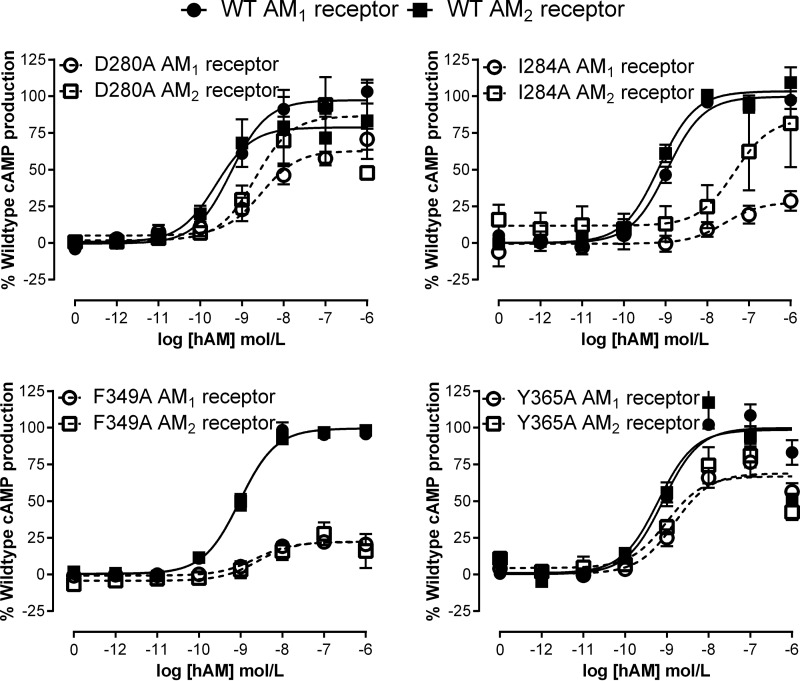
**Examples of mutants with common effects on cAMP production in both the AM_1_ and AM_2_ receptors.** Concentration-response curves are combined normalized data ± S.E. (*error bars*) for at least three individual experiments.

**FIGURE 5.**
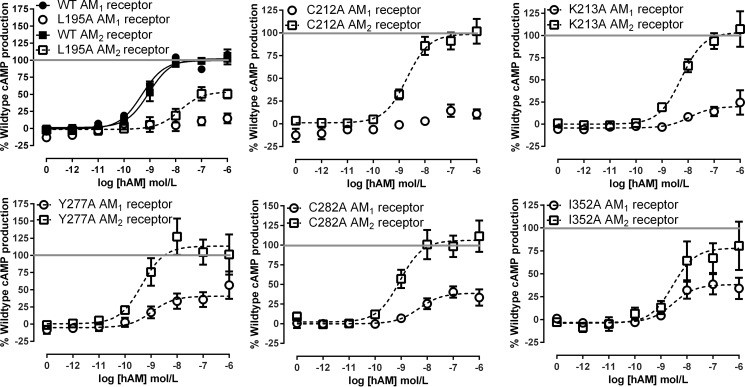
**Examples of mutants with common-differential and differential (C282A and Y277A) effects on cAMP production between the AM receptors.** WT curves were included in every experiment but are only shown as examples for L195A so that mutant differences between the receptors are not obscured by these curves in the other *panels*. The *horizontal line* represents maximal (100%) cAMP accumulation for the WT receptors. Concentration-response curves are combined normalized data ± S.E. (*error bars*) for at least three individual experiments.

**FIGURE 6.**
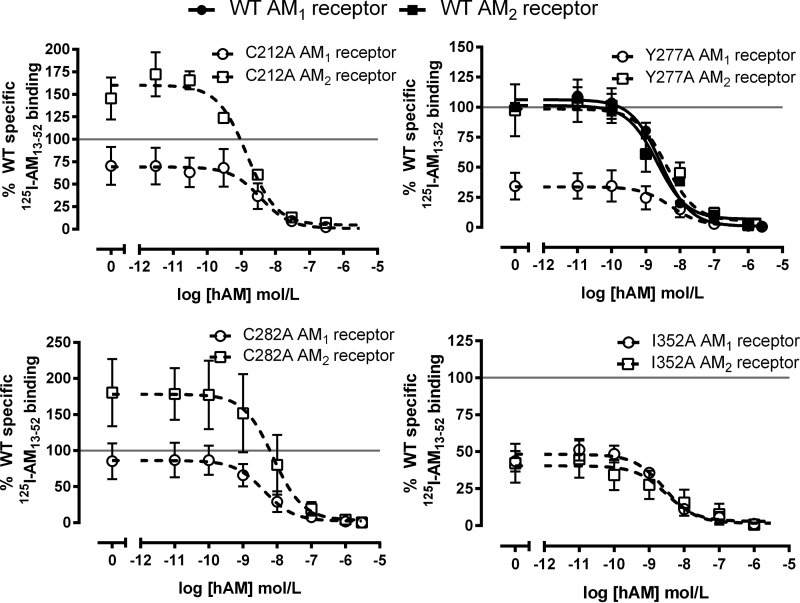
**^125^I-AM(13–52) binding at selected mutants with common-differential and differential (Y277A and C282A) effects in cAMP assays.** The curves are combined normalized data ± S.E. (*error bars*) for three individual experiments.

We assayed a total of 68 CLR mutants with RAMP2 and with RAMP3. All results are reported in Tables 2 and 3. cAMP data for selected mutants, which illustrate a breadth of effects, are shown in Figs. 4 and 5. The mutations could in principle change either the affinity of binding of AM or its ability to activate the receptor (efficacy). Efficacy can be estimated to some extent from *E*_max_, but this is limited by receptor reserve. Furthermore, for many mutants, we cannot measure affinity directly because the only radioligand available to us is the agonist, ^125^I-AM, which will not give detectable binding once its affinity goes below around 10 nm. The EC_50_ describes potency but does not provide a ready means for identifying mutants that alter efficacy as well as affinity. Accordingly, we have used Δlog(RA) (see “Experimental Procedures”) as a simple parameter to characterize the effect of the mutations in functional assays; where appropriate, we supplement this with observations on *E*_max_ or EC_50_. Using this, we describe below our major observations, categorized according to the effect of the mutation. We have also conducted radioligand binding assays using ^125^I-AM on selected mutants to provide additional information (Fig. 6).

**TABLE 2:** **Pharmacological parameters of cAMP accumulation for the AM receptors when stimulated by AM** *, *p* < 0.05; **, *p* < 0.01; ***, *p* < 0.001 *versus* WT, by unpaired *t* test except for Δlog(RA), where the comparison is between AM_1_ and AM_2_ receptors by two-way analysis of variance followed by Sidak's multiple comparison test. Common residues are in boldface type, common-differential residues are in boldface italic type, and differential residues are in italic type.

	AM_1_ receptor						AM_2_ receptor					
	WT pEC_50_	Mutant pEC_50_	ΔLog pEC_50_	*E*_max_ (%WT)	ΔLog(RA)	*n*	WT pEC_50_	Mutant pEC_50_	ΔLog pEC_50_	*E*_max_ (%WT)	ΔLog(RA)	*n*
**TM2**												
***L195A***	9.28 ± 0.10	<6	>2.00	No curve*^a^*	-	5	9.11 ± 0.16	7.40 ± 0.17***	1.71	65.3 ± 11.3*	1.90 ± 0.25*^b,c^*	5
T196A	8.94 ± 0.13	8.95 ± 0.03	−0.01	119.5 ± 23.5	−0.09 ± 0.16	3	8.90 ± 0.19	8.66 ± 0.19	0.24	116.5 ± 20.4	0.17 ± 0.28	3
A197L	8.91 ± 0.25	8.61 ± 0.13	0.30	90.4 ± 6.32	0.34 ± 0.28	4	9.19 ± 0.14	8.90 ± 0.11	0.29	136.9 ± 30.1	0.15 ± 0.20	3
***V198A***	9.13 ± 0.11	8.32 ± 0.15***	0.81	74.5 ± 12.2	0.94 ± 0.20*^b^*	7	9.41 ± 0.14	8.64 ± 0.19*	0.77	160.6 ± 26.16*	0.56 ± 0.25	3

**ECL1**												
**A199L**	9.14 ± 0.12	8.10 ± 0.23**	1.04	55.4 ± 8.56**	1.29 ± 0.28*^b^*	6	9.29 ± 0.16	7.98 ± 0.20**	1.31	88.6 ± 9.46	1.36 ± 0.26	4
N200A	9.11 ± 0.16	9.55 ± 0.10	0.44	80.9 ± 17.4	−0.35 ± 0.21	3	9.16 ± 0.14	9.57 ± 0.22	−0.41	69.3 ± 3.75***	−0.25 ± 0.26	3
N201A	9.02 ± 0.16	8.70 ± 0.18	0.32	89.8 ± 4.70	0.37 ± 0.24	4	9.14 ± 0.10	8.50 ± 0.22	0.64	133.3 ± 36.3	0.52 ± 0.27	3
Q202A	9.00 ± 0.07	9.15 ± 0.09	−0.15	82.9 ± 11.1	−0.069 ± 0.13	4	9.14 ± 0.10	9.30 ± 0.13	−0.16	184.4 ± 40.3	−0.43 ± 0.19	3
A203L	9.27 ± 0.08	9.14 ± 0.08	0.13	82.5 ± 6.92*	0.21 ± 0.12	5	9.14 ± 0.10	9.14 ± 0.08	0.00	207.5 ± 57.6	−0.32 ± 0.17	3
L204A	8.93 ± 0.04	8.62 ± 0.03**	0.31	98.9 ± 5.36	0.31 ± 0.06	3	9.42 ± 0.29	8.89 ± 0.16	0.53	145.7 ± 24.9	0.37 ± 0.34	3
V205A	9.10 ± 0.06	8.67 ± 0.14*	0.43	79.9 ± 16.8	0.53 ± 30.18	4	9.42 ± 0.29	9.10 ± 0.10	0.32	135.2 ± 34.7	0.19 ± 0.32	3
A206L	9.30 ± 0.08	8.93 ± 0.09*	0.37	113.3 ± 17.8	0.32 ± 0.14	4	9.42 ± 0.29	9.33 ± 0.14	0.09	118.2 ± 43.4	0.02 ± 0.36	3
T207A	9.10 ± 0.06	9.08 ± 0.16	0.02	87.6 ± 2.59*	0.077 ± 0.17	4	9.41 ± 0.05	9.21 ± 0.05	0.20	81.7 ± 14.2	0.29 ± 0.10	4
N208A	8.97 ± 0.06	8.73 ± 0.10	0.24	84.9 ± 10.7	0.31 ± 0.13	5	9.41 ± 0.05	8.82 ± 0.04***	0.59	105.4 ± 39.8	0.57 ± 0.18	4
P209A	8.99 ± 0.02	8.63 ± 0.09**	0.36	109.0 ± 13.4	0.32 ± 0.11	4	9.41 ± 0.05	8.88 ± 0.15*	0.53	107.2 ± 40.2	0.50 ± 0.23	4
V210A	9.19 ± 0.06	9.03 ± 0.14	0.16	74.9 ± 5.17**	0.29 ± 0.16	4	9.25 ± 0.10	9.03 ± 0.08	0.22	133.3 ± 18.9	0.10 ± 0.14	3
S211A	9.09 ± 0.10	8.97 ± 0.11	0.12	103.5 ± 12.1	0.11 ± 0.15	5	9.25 ± 0.10	9.10 ± 0.03	0.15	143.8 ± 66.6	−0.01 ± 0.23	3

**TM3**												
***C212A***	9.04 ± 0.21	<6	>2.00	No curve	-	6	9.20 ± 0.07	8.59 ± 0.15**	0.61	91.51 ± 18.7	0.65 ± 0.19*^c^*	6
***K213A***	9.22 ± 0.09	8.05 ± 0.09***	1.17	21.2 ± 7.38***	1.84 ± 0.204*^b^*	5	9.14 ± 0.08	8.31 ± 0.07***	0.83	97.7 ± 8.43	0.84 ± 0.11*^b^*	5
V214A	9.11 ± 0.12	9.01 ± 0.17	0.10	89.4 ± 7.11	0.15 ± 0.21	5	9.14 ± 0.11	9.14 ± 0.17	0.00	98.7 ± 9.12	0.01 ± 0.21	4
S215A	8.90 ± 0.12	8.81 ± 0.06	0.09	99.3 ± 1.87	0.093 ± 0.13	4	9.21 ± 0.12	9.22 ± 0.21	0.01	111.4 ± 13.8	−0.06 ± 0.25	3
Q216A	9.00 ± 0.12	9.60 ± 0.24	−0.6	100.2 ± 20.1	−0.60 ± 0.28	5	9.45 ± 0.14	9.42 ± 0.15	0.03	84.9 ± 7.41	0.10 ± 0.21	3
F217A	9.19 ± 0.03	8.82 ± 0.11**	0.37	97.3 ± 18.8	0.38 ± 0.14	7	9.41 ± 0.11	8.99 ± 0.15	0.44	100.3 ± 17.0	0.42 ± 0.20	5

**TM4**												
*A271L*	9.27 ± 0.14	8.33 ± 0.23**	0.94	71.4 ± 9.83*	1.09 ± 0.28	5	9.41 ± 0.09	9.16 ± 0.18	0.25	98.8 ± 33.7	0.26 ± 0.25	5
I272A	9.31 ± 0.18	9.65 ± 0.18	−0.34	151.6 ± 87.8	−0.52 ± 0.36	6	9.41 ± 0.09	9.42 ± 0.08	0.01	94.3 ± 18.1	0.02 ± 0.15	5
A273L	8.96 ± 0.14	8.91 ± 0.09	0.05	147.4 ± 42.7	−0.12 ± 0.215	4	9.26 ± 0.10	9.15 ± 0.09	0.11	100.4 ± 9.90	0.11 ± 0.14	4
***R274A***	9.51 ± 0.18	7.32 ± 0.14***	2.19	17.7 ± 5.01***	2.94 ± 0.26*^b^*	5	9.24 ± 0.08	8.24 ± 0.11***	1.00	31.6 ± 10.1***	1.50 ± 0.19^b^***	5

**ECL2**												
S275A	9.29 ± 0.13	9.39 ± 0.21	−0.10	103.3 ± 18.2	−0.11 ± 0.26	5	9.30 ± 0.15	9.23 ± 0.22	−0.10	114.9 ± 22.6	0.01 ± 0.28	5
L276A	9.29 ± 0.17	9.23 ± 0.15	0.06	96.8 ± 18.0	0.074 ± 0.24	4	9.30 ± 0.15	9.34 ± 0.16	−0.04	70.2 ± 12.4	0.11 ± 0.23	4
*Y277A*	9.51 ± 0.13	8.79 ± 0.19*	0.72	33.3 ± 8.98***	1.20 ± 0.26*^b^*	7	9.35 ± 0.09	9.23 ± 0.05	0.12	113.7 ± 21.2	0.06 ± 0.13**	5
*Y278A*	9.54 ± 0.13	8.45 ± 0.13***	1.13	55.9 ± 9.33***	1.34 ± 0.20*^b^*	7	9.34 ± 0.12	8.92 ± 0.09	0.42	106.0 ± 29.5	0.39 ± 0.19	5
*N279A*	9.04 ± 0.10	8.53 ± 0.31	0.25	21.7 ± 25.9	1.17 ± 0.614	4	9.45 ± 0.10	9.17 ± 0.10	0.28	95.6 ± 11.8	0.30 ± 0.15	4

**D280A**	9.29 ± 0.08	8.39 ± 0.08***	0.90	73.6 ± 7.61**	1.03 ± 0.12*^b^*	6	9.56 ± 0.07	8.31 ± 0.26**	1.25	93.7 ± 19.0	1.28 ± 0.28*^b^*	5
N281A	9.32 ± 0.08	9.26 ± 0.10	0.06	128.7 ± 16.9	−0.05 ± 0.14	5	9.64 ± 0.10	9.44 ± 0.19	0.20	143.9 ± 16.7	0.04 ± 0.22	4
*C282A*	9.09 ± 0.05	8.15 ± 0.21**	0.94	48.5 ± 10.2***	1.25 ± 0.23*^b^*	6	9.10 ± 0.11	8.88 ± 0.18	0.22	117.3 ± 16.7	0.15 ± 0.22*	5
***W283A***	9.19 ± 0.13	6.96 ± 0.17***	2.23	25.9 ± 11.7***	2.82 ± 0.29*^b^*	5	9.54 ± 0.08	7.96 ± 0.10***	1.58	71.7 ± 27.5	1.72 ± 0.21^b^*	5

**I284A**	8.97 ± 0.11	6.75 ± 0.35**	2.02	35.2 ± 7.25***	2.67 ± 0.38*^b^*	5	9.08 ± 0.12	7.36 ± 0.21***	1.72	51.8 ± 18.4**	2.01 ± 0.29*^b^*	5
S285A	9.33 ± 0.07	9.02 ± 0.08	0.31	196.7 ± 113.9	0.02 ± 0.27	4	9.60 ± 0.09	9.37 ± 0.11	0.23	157.7 ± 52.8	0.03 ± 0.20	5
S286A	9.16 ± 0.27	9.31 ± 0.20	−0.15	99.2 ± 16.8	−0.15 ± 0.34	4	9.45 ± 0.18	9.66 ± 0.11	−0.19	71.4 ± 26.4	−0.06 ± 0.26	3
D287A	9.26 ± 0.05	9.51 ± 0.47	−0.25	105.3 ± 34.5	−0.27 ± 0.49	4	9.31 ± 0.05	9.12 ± 0.12	0.19	104.5 ± 18.8	0.17 ± 0.15	4

**T288A**	9.15 ± 0.13	8.37 ± 0.05***	0.78	51.4 ± 3.32***	1.07 ± 0.14*^b^*	4	9.60 ± 0.09	8.97 ± 0.11***	0.63	78.46 ± 14.6	0.74 ± 0.16	4
H289A	9.09 ± 0.04	9.12 ± 0.13	−0.03	118.7 ± 8.74	−0.10 ± 0.14	4	9.58 ± 0.26	9.74 ± 0.25	−0.16	298.8 ± 143.9	−0.64 ± 0.42	5
L290A	8.96 ± 0.14	8.83 ± 0.09	0.13	190.9 ± 84.2	−0.15 ± 0.25	4	9.26 ± 0.09	8.97 ± 0.08	0.29	94.4 ± 13.8	0.32 ± 0.14	3
L291A	9.09 ± 0.04	8.62 ± 0.14*	0.47	136.7 ± 45.1	0.33 ± 0.20	4	9.18 ± 0.12	8.83 ± 0.15	0.35	125.1 ± 32.2	0.25 ± 0.22	6

**TM5**												
Y292A	8.96 ± 0.14	8.54 ± 0.06*	0.42	91.2 ± 18.9	0.46 ± 0.18	4	9.32 ± 0.10	8.67 ± 0.17*	0.65	135.1 ± 42.2	0.52 ± 0.24	4
I293A	8.97 ± 0.04	9.20 ± 0.07*	−0.28	93.2 ± 29.6	−0.20 ± 0.16	4	9.01 ± 0.07	9.13 ± 0.02	−0.12	121.8 ± 23.1	−0.21 ± 0.11	3
I294A	9.09 ± 0.04	8.83 ± 0.21	0.26	147.5 ± 53.5	0.09 ± 0.26	4	9.18 ± 0.12	9.12 ± 0.14	0.06	128.3 ± 19.1	−0.05 ± 0.19	5

**TM6**												
**F349A**	9.01 ± 0.11	8.72 ± 0.11	0.29	21.5 ± 1.81***	0.96 ± 0.16*^b^*	5	9.14 ± 0.05	8.61 ± 0.07**	0.53	24.0 ± 9.13***	1.15 ± 0.19*^b^*	5
V350A	8.86 ± 0.28	8.72 ± 0.07	0.14	63.7 ± 4.94***	0.34 ± 0.29	4	9.14 ± 0.05	8.87 ± 0.34	0.27	65.8 ± 17.3	0.45 ± 0.36	4
L351A	9.15 ± 0.10	No curve	>3	No Curve	-	4	9.09 ± 0.06	No Curve	>3	No Curve	-	3
***I352A***	9.01 ± 0.11	8.28 ± 0.27*	0.73	37.7 ± 9.87***	1.15 ± 0.31	5	9.40 ± 0.08	8.46 ± 0.10***	0.94	86.9 ± 30.1	1.00 ± 0.20*^b^*	5
***P353A***	9.07 ± 0.13	No Curve	>3	No Curve	-	5	9.42 ± 0.09	8.49 ± 0.06***	0.91	47.1 ± 9.19***	1.26 ± 0.14*^b,c^*	5
***W354A***	9.29 ± 0.06	8.50 ± 0.12***	0.79	37.2 ± 7.38***	1.22 ± 0.16*^b^*	5	9.40 ± 0.08	8.59 ± 0.13***	0.81	112.4 ± 17.5	0.76 ± 0.17*^b^*	5
R355A	9.13 ± 0.08	9.50 ± 0.12*	−0.37	80.4 ± 7.97*	−0.27 ± 0.15	4	9.27 ± 0.26	9.89 ± 0.03	−0.62	91.0 ± 18.6	−0.58 ± 0.28	3
P356A	9.15 ± 0.10	8.99 ± 0.10	0.16	40.4 ± 3.66***	0.55 ± 0.15	4	9.34 ± 0.20	9.12 ± 0.29	0.22	60.0 ± 23.1	0.44 ± 0.39	4

**ECL3**												
E357A	9.00 ± 0.08	No Curve	>3	No Curve	-	4	9.23 ± 0.26	No Curve	>3	No Curve	-	3
G358A	9.09 ± 0.11	9.06 ± 0.11	0.03	104.5 ± 5.46	0.01 ± 0.16	4	8.79 ± 0.14	8.84 ± 0.27	−0.05	80.9 ± 55.3	0.04 ± 0.42	3
K359A	9.09 ± 0.11	9.19 ± 0.10	−0.10	107.5 ± 4.28	−0.13 ± 0.15	4	9.27 ± 0.26	9.30 ± 0.14	−0.03	75.9 ± 4.14***	0.09 ± 0.29	3
I360A	9.13 ± 0.18	8.99 ± 0.08	0.14	83.3 ± 9.47	0.22 ± 0.20	4	8.79 ± 0.14	8.79 ± 0.29	0.00	82.8 ± 35.9	0.08 ± 0.37	3
***A361L***	9.10 ± 0.18	9.52 ± 0.15	−0.42	77.8 ± 6.34*	−0.31 ± 0.24	4	9.15 ± 0.13	8.62 ± 0.40	0.53	67.4 ± 12.4*	0.70 ± 0.43	4
E362A	9.10 ± 0.18	9.00 ± 0.07	0.10	98.0 ± 6.53	0.11 ± 0.19	4	9.22 ± 0.10	8.91 ± 0.10	0.21	241.7 ± 76.1	−0.07 ± 0.20	3
E363A	9.00 ± 0.08	8.87 ± 0.15	0.13	93.9 ± 13.5	0.16 ± 0.18	4	9.22 ± 0.10	8.99 ± 0.11	0.23	217.7 ± 72.3	−0.11 ± 0.21	3
V364A	9.00 ± 0.08	8.84 ± 0.13	0.16	84.3 ± 8.49	0.23 ± 0.16	4	9.16 ± 0.09	9.14 ± 0.14	0.02	83.2 ± 3.57	0.10 ± 0.17	4

**TM7**												
**Y365A**	9.09 ± 0.11	8.83 ± 0.11	0.26	68.9 ± 6.54**	0.42 ± 0.16	4	9.02 ± 0.12	8.51 ± 0.33	0.51	65.2 ± 0.85**	0.70 ± 0.35	3
D366A	8.97 ± 0.08	8.76 ± 0.15	0.21	69.9 ± 15.7	0.37 ± 0.19	4	9.17 ± 0.11	9.19 ± 0.30	−0.02	105.7 ± 23.8	−0.04 ± 0.33	3
*Y367A*	9.16 ± 0.10	9.11 ± 0.03	0.04	70.0 ± 6.69	0.20 ± 0.11	3	9.28 ± 0.17	8.61 ± 0.16*	0.67	163.7 ± 32.8	0.46 ± 0.25	3
I368A	9.06 ± 0.12	9.06 ± 0.08	0.00	77.3 ± 12.6	0.11 ± 0.16	4	9.28 ± 0.17	9.39 ± 0.25	−0.11	251.1 ± 120	−0.51 ± 0.37	3
M369A	9.06 ± 0.12	9.06 ± 0.13	0.00	185.1 ± 28.0*	−0.27 ± 0.19	4	9.28 ± 0.17	8.82 ± 0.27	0.46	295.7 ± 103.1	−0.01 ± 0.35	3

*^a^* No curve, cAMP response was too low for a concentration-response curve to be fitted (pEC_50_ and Δlog pEC_50_ are denoted as <6 and >2).

*^b^* Different from 0, as assessed by multiple t tests with the false discovery rate set to 1%.

*^c^* ΔLog(RA) values where only the AM_2_ receptor was active.

**TABLE 3:** **Pharmacological parameters for ^125^I-AM(13–52) binding for WT or mutant AM receptors** Common-differential residues are ***bold italics*** and differential residues are *italics*. Maximum specific binding is total binding (^125^I-AM_13–52_ bound in the absence of competing ligand) minus the non-specific binding (^125^I-AM_13–52_ bound in the presence of 3 μm AM).

	AM_1_ receptor			AM_2_ receptor		
	pIC_50_	Maximum specific binding (%WT)	*n*	pIC_50_	Maximum specific binding (%WT)	*n*
WT	8.56 ± 0.04		4	8.64 ± 0.07		4
***C212A***	8.06 ± 0.16	71.7 ± 20.7	3	8.35 ± 0.18	145.5 ± 23.4	3
*Y277A*	8.46 ± 0.35	34.3 ± 11.1*^a^*	3	8.52 ± 0.15	97.4 ± 21.6	3
*C282A*	8.40 ± 0.15	85.4 ± 24.9	3	8.31 ± 0.29	180.4 ± 46.6	3
***I352A***	8.56 ± 0.10	43.5 ± 7.0*^a^*	3	8.65 ± 0.21	42.2 ± 13.2*^a^*	3

*^a^* 95% confidence interval does not include 100%.

There was a core subset of six residues that were important for the function of the AM_1_ and AM_2_ receptors (Ala^199^, Asp^280^, Ile^284^, Thr^288^, Phe^349^, and Tyr^365^), producing shared changes in pEC_50_, *E*_max_ or Δlog(RA). We define all of these six as having common effects (Fig. 4). These residues are situated within ECL2 and the TM6-ECL3-TM7 juxtamembrane region, along with A199L in TM2.

A further 10 mutations had an effect at both AM receptors, but the nature of the effect differed between the two receptors (Leu^195^, Val^198^, Cys^212^, Lys^213^, Arg^274^, Trp^283^, Ile^352^, Pro^353^, Trp^354^, and Ala^361^). These are defined as residues with common but differential effects (Fig. 5). L195A in TM2, C212A at the ECL1-TM3 boundary, and P353A at the TM6-ECL3 boundary abolished AM-mediated cAMP production at the AM_1_ receptor, whereas K213A reduced this by 80%. For C212A, there was a trend for the radioligand binding to be modestly reduced at the AM_1_ receptor but enhanced at the AM_2_ receptor, consistent with a differential effect of this mutation at both receptors (Fig. 6). The corresponding mutations in the AM_2_ receptor were less deleterious. I352A and W354A mutations gave very similar changes in Δlog(RA), and radioligand binding shows a similar reduction in specific binding for I352A at both receptors (Fig. 6). However, in both cases, the effects on *E*_max_ were more marked at the AM_1_ receptor, so these have been included as common but differential residues. Whereas V198A showed only a small difference in Δlog(RA), it significantly increased *E*_max_ at the AM_2_ receptor but not the AM_1_ receptor. A361L was a difficult mutant to characterize; whereas the *E*_max_ is reduced at both the AM_1_ and AM_2_ receptors, the changes in Δlog(RA) were of opposing directions.

Five of the 68 mutants had more pronounced differential effects between the receptors. These are referred to as differential residues (Fig. 5). A271L, Y277A, Y278A, N279A, and C282A all increased Δlog(RA) at the AM_1_ receptor but had no significant effect at the AM_2_ receptor. For Y277A, radioligand binding was substantially reduced at the AM_1_ receptor but retained at the AM_2_ receptor, consistent with a differential effect of this mutation at both receptors. C282A binding was unchanged at the AM_1_ receptor but showed a trend to be enhanced at the AM_2_ receptor (Fig. 6). In addition, for Y367A, we observed a decrease in pEC_50_ at the AM_2_ receptor but no effect at the AM_1_ receptor. Although the differences in *E*_max_ at either receptor did not reach statistical significance, the effect was opposite with an increase at the AM_2_ receptor and a decrease at the AM_1_ receptor. This is an atypical mutation because the effect is greater at the AM_2_ receptor.

##### Overall Description of the AM_1_ and AM_2_ Receptor Models

**FIGURE 7.**
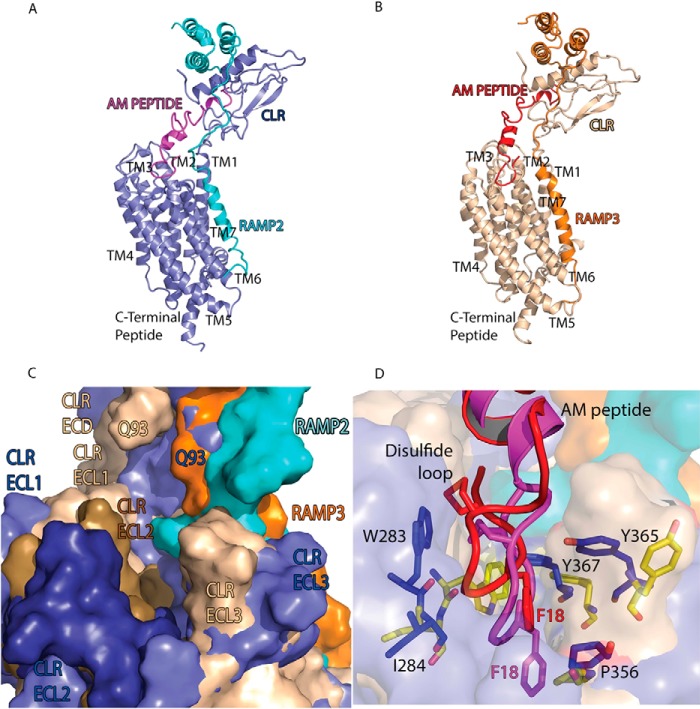
**Models of the full-length AM receptors.** *A*, AM_1_ receptor; *B*, AM_2_ receptor. Images were generated from an overlay aligning CLR residues 138–394 for both models (root mean square deviation = 2.0 Å). Relative sizes and orientations are thus not an artifact of figure generation. *C*, *surface representation* of the peptide binding pocket of the AM_1_ and AM_2_ receptors illustrating the changes in receptor conformation and the peptide binding pocket. *D*, *close-up surface representation* of the peptide binding pocket showing the docked AM peptide and its five close receptor neighbors, determined by the models in *blue sticks* (AM_1_ receptor) and *yellow sticks* (AM_2_ receptor). *Other colors* in *C* and *D* are as described for *A* and *B*.

To assist in data interpretation, we generated AM_1_ and AM_2_ receptor models, which we understand to be the first models of a full-length GPCR in complex with a RAMP (Fig. 7, *A* and *B*). The RAMP TM helix lies between TM6 and TM7 of CLR without inducing strain in the sequence joining the RAMP ECD to the TM (the RAMP linker). The predicted arrangement of the TM helices forms a conical pocket (the peptide binding site) into which the disulfide loop of the AM peptide docks (Fig. 7, *C* and *D*). ECL boundaries are very similar to those in the CGRP receptor (26) and those of other class B GPCR x-ray structures (43, 50).

In the AM peptide model, residues 15–21 form a disulfide loop, residues 22–31 are helical (40), and residues 35–52 adopt the largely non-helical structure bound to the ECD of the AM_1_ receptor (21); the remaining residues (positions 33–41) form a loop, creating the AM structure. The model therefore rationalizes previous work on the degree of helicity within AM (Fig. 1*E*). The RAMP2 linker (residues Val^134^–Leu^147^ between the ECD and the TM region) is displaced relative to that of RAMP3, lies closer to the peptide binding pocket than does RAMP3, and is predicted by the models to interact with ECL3 and the top of TM7 of CLR (Figs. 7 and 9).

**FIGURE 8.**
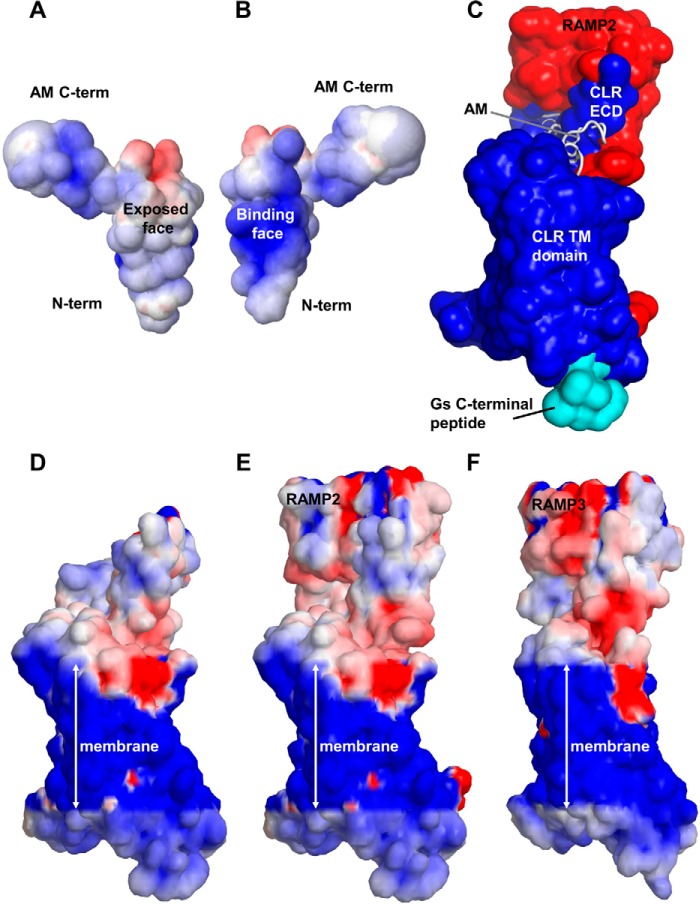
**The electrostatic potential of AM, CLR, and the AM_1_ and AM_2_ receptors.** *Blue*, positive; *red*, negative; the potential has been contoured between −5 and +5 onto the solvent-accessible surface. *A*, the electrostatic potential on the face of AM that is exposed as it binds to the CLR ECD; the electrostatic potential is weakly positive, and the general orientation is as shown in *C. B*, the electrostatic potential on the ECD-binding surface, as defined by *C*; the electrostatic potential is strongly positive. The +4 charge on AM includes the N-terminal amine. *C*, a representation of AM binding to the AM_1_ receptor that can be used to identify the peptide-binding region in *D–F*: the CLR solvent-accessible surface is *blue*, the RAMP surface is *red*, and the C-terminal peptide of G_s_ is *cyan*. The solvent radius was expanded to 2.4 Å to mimic that in *D–F*. AM is shown in *white. D*, CLR electrostatic potential. *E*, AM_1_ electrostatic potential. *F*, AM_2_ electrostatic potential. The electrostatic potential of the AM_1_ and AM_2_ receptors was evaluated in an implicit membrane using APBS (the Adaptive Poisson-Boltzmann Solver) coupled with apbs_mem version 2.0 and the pdb2PQR server (74–76). The parameters for the APBSmem calculations were as follows: PARSE atomic charges (77); temperature, 298.15 K; ionic strength, 0.15 mm; protein and membrane relative dieletric constant, 2.0; relative solvent dielectric, 80; membrane thickness, 40 Å. The grid lengths were 300 × 300 × 300 Å with two levels of focusing; the grid dimensions were 97 × 97 × 97 for *A* and *B* and 129 × 129 × 225 for *D–F*. The CHARMM-gui was used to assist in placing the receptor within the membrane (78).

The electrostatic potential of AM in its proposed bound conformation (Fig. 8, *A* and *B*) is largely positive because AM carries a charge of +4. The electrostatic potential of CLR in the absence of RAMP and AM is largely positive or neutral (Fig. 8, *C* and *D*). Both RAMP2 and RAMP3 convey an advantage in binding the positive AM because they switch this potential in the conical TM pocket and particularly on the ECD to more negative values, which will aid in binding the positively charged AM (Fig. 8, *E* and *F*). RAMP3 gives rise to the most negative ECD electrostatic potential.

##### Detailed Comparison between AM_1_ and AM_2_ Receptor Models

**FIGURE 9.**
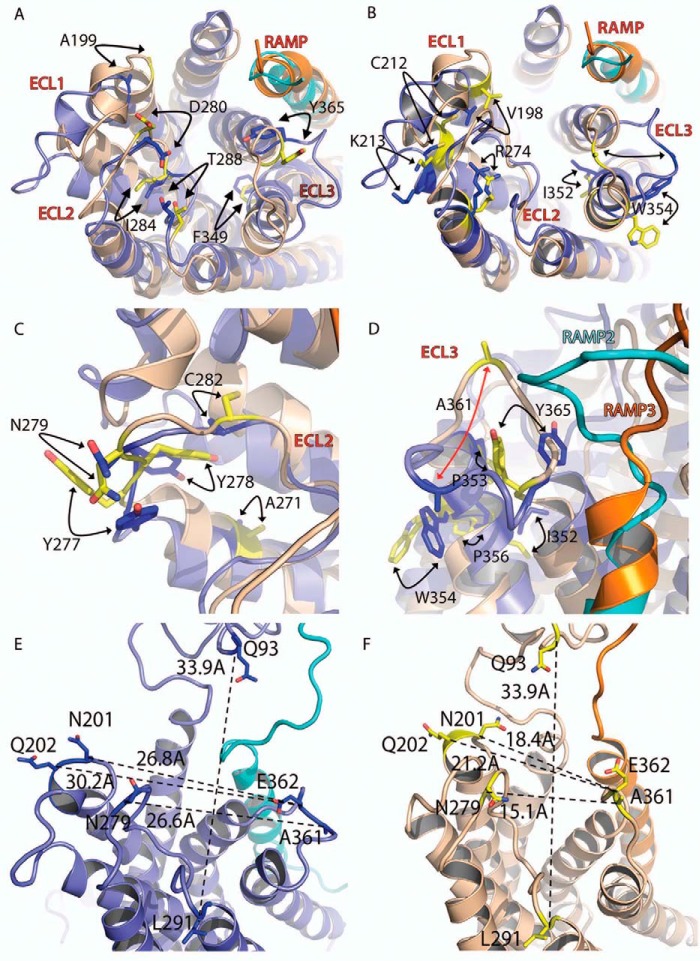
**Receptor model overlay.** Residues with common (*A*), common-differential (*B*), or differential (*C*) effects are shown as *sticks*, with oxygen atoms in *red* and nitrogen in *blue. A*, residues Ala^199^, Asp^280^, Ile^284^, and Phe^349^ have similar side chain and main chain orientations; Tyr^365^ has side chain rotation of ∼180° between the two AM receptors. *B*, residues Lys^213^, Ile^352^, and Trp^354^ have similar main chain but differing side chain orientations. *C*, Tyr^277^ shows substantial movement between the two receptors, whereas Tyr^278^ shows some movement but maintains similar interactions. *D*, *close-up view* of TM6-ECL3-TM7 showing the main residues involved in the change of the ECL3 position (*red arrow* denotes change in position). The increased proximity of RAMP2 to the CLR ECL3 in the AM_1_ receptor is clearly visible. AM_1_ and AM_2_ receptors are *colored* as per the figure; the movement of residues between the receptors is shown with *arrows. E* and *F*, juxtamembrane region of the two receptors with distances between residues (*dotted lines*) in Å. Distances were measured between the same set of Cα atoms in both receptors.

Overall, the ECL2 conformation is similar between the two models, consistent with the observation that many of the residues with common cAMP effects are located in this invariant region and may contact the peptide (Fig. 9*A*). Residues with common but differential effects at each receptor also have largely similar orientations within the models (Fig. 9*B*). These residues are also mostly situated at the tops of the TM2 (Leu^195^ and Val^198^), TM3 (Cys^212^ and Lys^213^), and TM6 (Ile^352^, Pro^353^, and Trp^354^). Along with the common residues Ala^199^ and Phe^349^ and common but differential Arg^274^, these form a network around the top of the TM helices. Differential residues Tyr^277^ and Cys^282^ are situated in ECL2 (Fig. 9*C*). Cys^212^, Tyr^278^, Cys^282^, and Lys^213^ do not appear to change their orientation significantly between the two AM receptors (Fig. 9, *B* and *C*). Lys^213^ remains parallel to the Cys^212^–Cys^282^ bond, facing Tyr^278^ in both structures. Tyr^277^ moves outward in the AM_2_ receptor and points away from the peptide binding pocket, thus changing its environment (Fig. 9*C*).

The most striking conformational difference between the AM_1_ and AM_2_ receptor models is the dramatic change in the position of ECL3 (Fig. 9*D*). The extracellular end of TM6 forms a distorted helix as a result of the influence of Pro^353^, Pro^356^, and Gly^358^. The conformation of ECL3 begins to diverge between the two models after the common differential residue Pro^353^. Trp^354^ stacks with ECL3 in the AM_1_ receptor, whereas in the AM_2_ receptor it is rotated by 90°, moving it away from the loop to face the lipid membrane. In the AM_2_ receptor model, ECL3 makes extensive contacts with AM, whereas in the AM_1_ receptor, these contacts are minimal. The cumulative result of these differences is that distances relevant to the binding site vary in size (Fig. 9, *E* and *F*).

##### Probing the Model; Differential Peptide Contacts within the AM_1_ and AM_2_ Receptor TM Pockets

**FIGURE 10.**
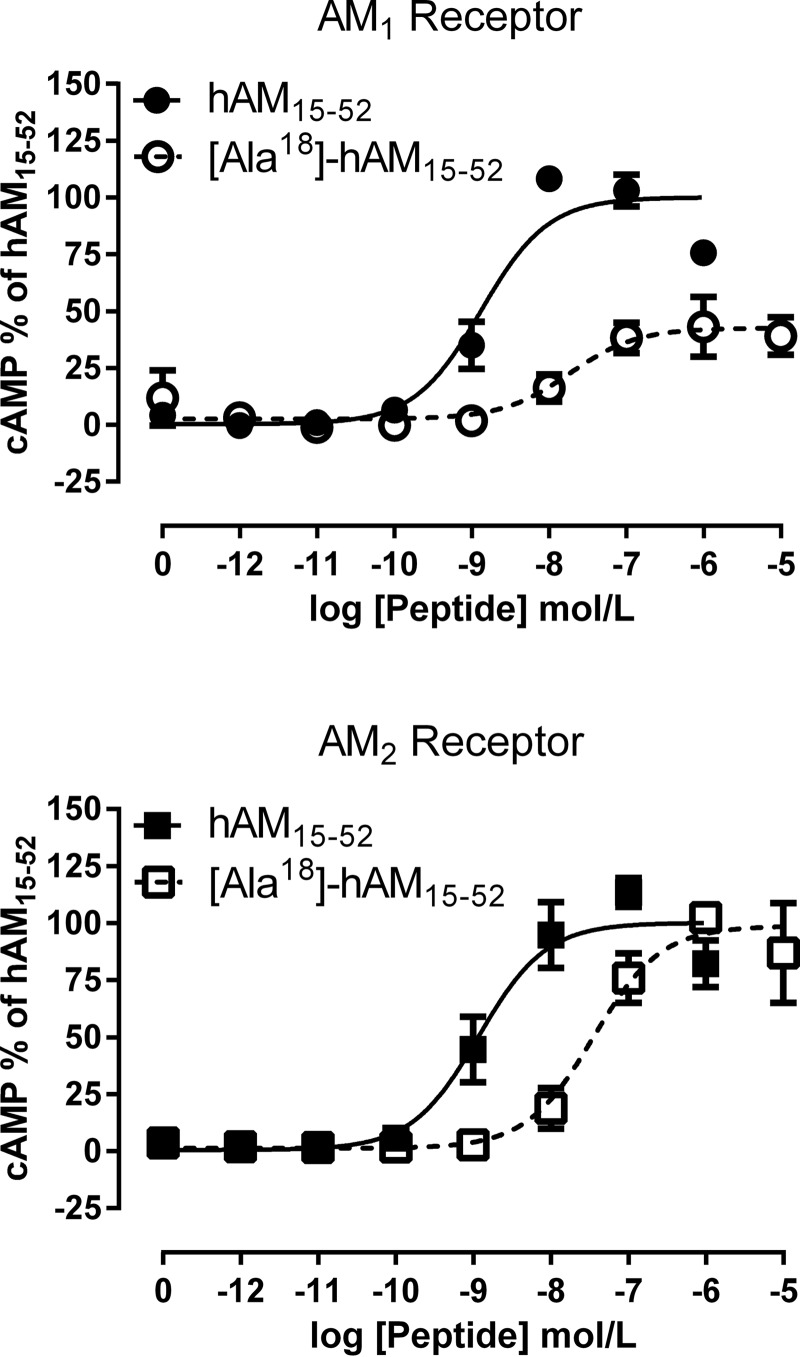
**Concentration-response curves for the alanine-substituted AM peptide, F18A AM(15–52).** Curves are combined normalized data from at least three individual experiments ± S.E. (*error bars*).

The divergence between the models translates into different transmembrane AM binding pocket hull volumes of 4874 Å^3^ for the AM_1_ receptor *versus* 3313 Å^3^ for the AM_2_ receptor; the shapes of the two pockets also differ. The disulfide loop (Cys^16^–Cys^21^) of the docked AM peptide is located in the wide mouth of the peptide binding pocket with the side chain of Phe^18^ occupying the lower part of the pocket (Fig. 7*D*). Visual analysis and loop modeling indicated that Phe^18^, unlike its neighbors, occupied a more constrained pocket in the AM_2_ receptor than in the AM_1_ receptor. Consequently, we examined R17A, F18A, G19A, and T20A mutations in both the AM_1_ and AM_2_ receptors using MODELER; 100 models were generated, and the model with the best DOPE score was analyzed. In each case, apart from F18A, there was an equivalent decrease in the number of contacts (<4 Å) in both AM_1_ and AM_2_, but for F18A, there was a bigger decrease in the number of side chain contacts in the AM_1_ receptor (from eight to two) rather than in the AM_2_ receptor (from six to two). We therefore proposed that substitution of Phe^18^ with alanine would have a greater impact in the AM_1_ receptor, compared with the AM_2_ receptor. Consistent with our hypothesis, an F18A AM peptide stimulated cAMP production to a lesser degree at the AM_1_ receptor (60% decrease in *E*_max_) than at the AM_2_ receptor (no change in *E*_max_) (Table 4 and Fig. 10). This demonstrates that it is possible to engineer ligand-specific effects at these two receptors.

**TABLE 4:** **Pharmacological parameters of cAMP accumulation for F18A substituted AM(15–52) versus wild type (WT) AM(15–52) stimulation of the WT AM_1_ and AM_2_ receptors** *, *p* < 0.05; ***, *p* < 0.001. Data analyzed by unpaired *t* test *versus* WT.

	WT AM(15–52) pEC_50_	F18A AM(15–52) pEC_50_	ΔLog EC_50_	% *E*_max_ WT AM(15–52)	*n*
AM_1_ receptor	8.89 ± 0.13	7.85 ± 0.11***	1.04	40.3 ± 9.73***	4
AM_2_ receptor	8.91 ± 0.24	7.42 ± 0.24*	1.49	101.7 ± 5.26	3

##### Small Molecule Druggability of the AM Receptors

**FIGURE 11.**
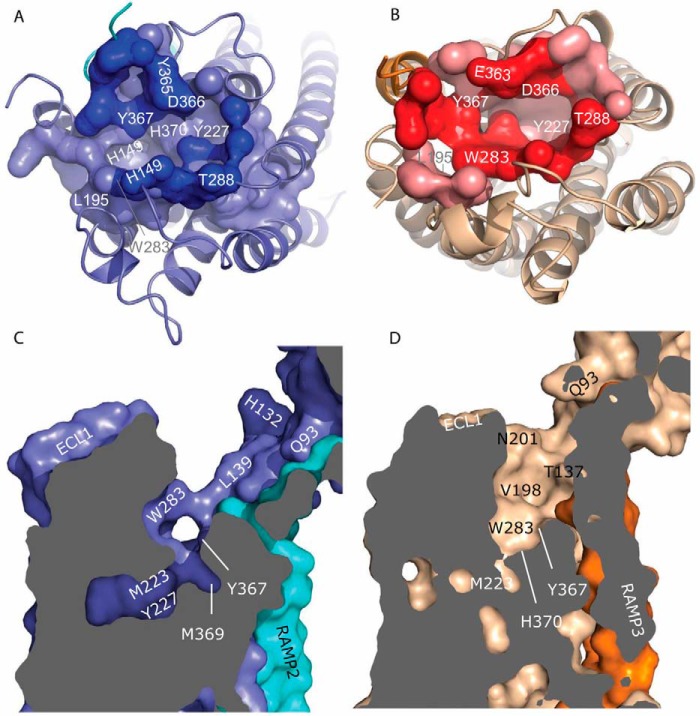
**Small molecule druggable sites predicted using PockDrug and viewed from above.** *A*, the AM_1_ site is shown in *light blue*, and the site residues that contact AM are shown in *blue. B*, the AM_2_ site is shown in *magenta*, and the site residues that contact AM are shown in *red*. This site is narrower and deeper than the AM_1_ site; the PockDrug druggability scores for the AM_1_ and AM_2_ sites are 0.97 and 0.91, respectively. *C* and *D*, surface cutaway views of the receptors; the different size, conformation, and situation of the pockets are evident from the *shading*. Selected residues are *labeled*.

We next analyzed the two receptor binding pockets for their druggability for small molecule, orally bioavailable ligands using the PockDrug and DoGSiteScorer druggability servers (52–54), which were trained to predict pockets with promising properties for the design of small molecule druglike ligands. Because druggability analysis is highly dependent on the cavity detection (53), we only discuss residues predicted by both servers to reside in the main helical binding pocket, namely 43 residues common to the AM_1_ receptor pocket and 31 for the smaller AM_2_ receptor pocket; these consensus residues largely coincide with the largest subpocket given by DoGSiteScorer. This analysis showed that the main druggable pocket in the helical domain of each AM receptor partially overlapped with the peptide binding pocket identified by our models (Fig. 11, *A* and *B*). In both receptors, the druggable pocket includes the hydrophobic patch at the top of TM2 (*e.g.* Leu^195^), the distal residues of ECL2 (Trp^283^–Thr^288^), and residues on TM3 (*e.g.* Asp^366^, Tyr^367^, and His^370^). The druggable pockets extend below the limits of the peptide binding pocket and include Met^223^ and Tyr^227^ on TM3 for both receptors, but the AM_1_ pocket includes other TM3 residues (*e.g.* Leu^220^). The druggable pockets also extend lower on TM6 to include Ile^370^ and Ile^371^ for the AM_1_ receptor. The AM_1_ pocket includes more residues on TM1 (*e.g.* Thr^145^ and His^149^). Twenty-four residues were unique to the AM_1_ receptor, and seven were unique to the AM_2_ receptor, indicating that selectivity is possible. Some of the residues listed as part of the druggable pocket are more accessible than others (*e.g.* Phe^228^ in the AM_1_ receptor is not obviously accessible in the absence of induced fit, because it is partially shielded by Tyr^227^), but such residues may nevertheless be important in drug design. The AM_1_ receptor pocket reaches 14 Å below the top of ECL3 with drug scores of 0.97 and 0.81, from PockDrug and DoGSiteScorer, respectively. The AM_2_ receptor druggable pocket forms a narrow channel and is deeper (partly because of the ECL3 conformation), with PockDrug and DoGSiteScorer drug scores of 0.91 and 0.81, respectively; because the scores are above 0.5, both receptors are predicted to be druggable.

## Discussion

Pharmacological tools to help tease out the relative importance of each of the two AM receptors are needed, but it has not been apparent how to develop these because both receptors share the common GPCR, CLR. We report that RAMP2 and RAMP3 confer conformational variation in the CLR juxtamembrane region, yielding distinct binding pockets that may be tractable for the development of selective pharmacological tools and future drugs.

Our study combined extensive mutagenesis of CLR with independent modeling studies (*i.e.* not adjusted to enhance agreement with data tables) that allowed us to effectively interpret our complex data set. Recent crystallographic and modeling studies have generated a consensus conformation for the TM bundle of the class B GPCRs (26, 43, 50, 55). Crystallographic studies have so far, however, proved unsatisfactory for determining the structure for a complete class B GPCR or for the class B ECL conformation due to the inherent mobility of the loops. The only structural data on the arrangement of the ECD of a class B GPCR with respect to the TM bundle comes from an electron microscopy study of the GCGR, and this is necessarily low resolution (56). Although molecular models do not have the accuracy of x-ray structures, they are nevertheless useful for providing a framework against which experimental results can be considered. While it would be unwise to overinterpret any model, ours is largely consistent with the effects of the mutagenesis (Table 5) and also successfully predicted the activity of F18A AM.

**TABLE 5:**
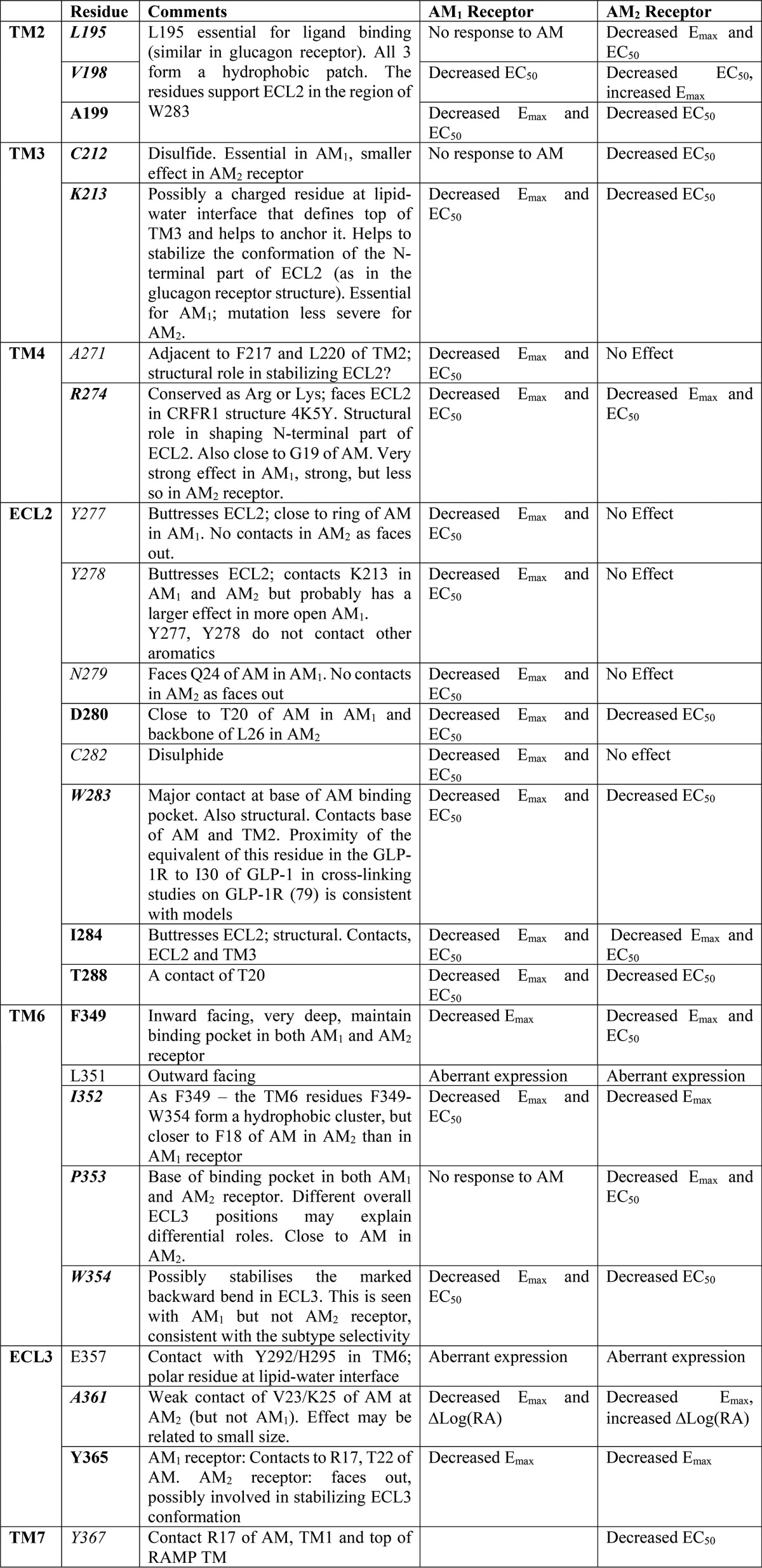
**Comments on the mutation data of residues discussed in this work and shown in Tables 1 and 2 in light of the receptor models** Common residues are shown in boldface type, common-differential residues are in boldface italic type, and differential residues are in italic type.

We initially compare our AM receptor models with that of the GCGR model structure (43), a canonical class B GPCR that does not require a RAMP. The main difference in the receptor is a ∼30° change in the orientation of the ECD to a more open conformation as a result of the constraint created by the RAMP on the structure of the AM receptor. The orientation of the ECD is more consistent with the open (*i.e.* active, agonist-bound) conformational ensemble of the GCGR (56) than the closed ensemble because the difference between the centers of mass of the TM and ECD domains is ∼57 Å, the polar angle θ is similarly ∼23°, and the projection of the ECD center of mass onto the membrane plane lies outside of the helical bundle. In the GCGR, the simulated closed state described by Yang *et al.* (56) may well be the inactive conformation, satisfying the proposed ECD-ECL3 interaction proposed by Koth *et al.* (57), but in the AM receptors, because the RAMP binds to the peptide-binding face of the ECD, it is likely to inhibit formation of the fully closed conformation. The peptide shows more marked differences: the glucagon model peptide adopts a helical structure from Ser^8^ through to Met^27^, spanning from the juxtamembrane region through to the ECD, in agreement with most x-ray crystal structures on isolated class B ECDs. In contrast, AM has a more complex structure, with a non-helical ECD region, in agreement with the x-ray crystal structure of the isolated ECD and a helical region that binds to the juxtamembrane region, as in previous related models and the AM NMR structure (40). The AM peptide helix binds to the same depth as the glucagon peptide, as judged by the alignment of the helical region (Fig. 1*A*), but the glucagon peptide N terminus binds to a greater depth (consistent with cross-linking data on the related PTH system (58), whereas AM forms a disulfide-bonded loop consistent not only with the binding of the usual AM(16–52) (26) but also AM(1–52) (*i.e.* the N terminus is orientated so that AM(1–15) can “escape” from the TM bundle). This N-terminal extension of AM does not seem important for AM activity, and the AM(15–52) fragment is more consistent with the length of other peptides in the AM family.

We have pharmacological evidence of RAMP-induced changes in the function of CLR at the AM_1_ and AM_2_ receptors, which are reflected in conformational differences between our full-length AM_1_ and AM_2_ receptor models. The most striking difference between the two models is the ECL3 conformation; interestingly, this is a region that also shows large differences between the GCGR and CRF1R x-ray crystal structures. Although only Ala^361^ in ECL3 shows any kind of differential activity, the residues flanking ECL3 do show this. Moreover, at the CGRP receptor, the CLR-RAMP1 complex, Ile^360^ is involved in receptor activation as opposed to Ala^361^ in the AM receptors (24), giving additional evidence of differential activity in ECL3. The extracellular region of TM6 in the AM receptors does contain residues with common-differential activity, namely Ile^352^, Pro^353^, and Trp^354^. The predicted stacking of Trp^354^ with ECL3 in the AM_1_ receptor combined with changes in the positions of Ile^352^ and Pro^353^ may stabilize the altered orientation of ECL3. In the AM_2_ receptor, Trp^354^ lies perpendicular to its AM_1_ receptor position, allowing ECL3 to lie further toward the center of the peptide binding pocket. Movement of the upper regions of TM3, TM6, and TM7 is involved in activation of class A GPCRs (59). Some of the differences observed between the two AM receptors could therefore be reflected in differential activity of residues in ECL3/TM7 (Ala^361^ and Tyr^367^) and TM3 (Cys^212^ and Lys^213^) in the two AM receptors and in the CGRP receptor, where Cys^212^ is the only one of these residues involved in receptor activation (24).

The AM model peptide interacts differently with ECL3/TM7 in the two AM receptors (Figs. 1 and 7, *A* and *B*) in response to the effect of the different RAMPs; our models place the RAMP TM helix between TM6 and TM7 as in the class B secretin receptor (60). The greater proximity of the RAMP2 ECD-TM linker to ECL3 is probably the main factor that contributes to the reorientation of ECL3 (Fig. 9*D*). RAMP2 and RAMP3 diverge in sequence in this region, and equivalent RAMP residues take up different positions relative to AM in the two models.

The majority of the residues with a common or a common but differential effect on receptor activation vary little in their orientation and cluster around the upper TMs of our models (*e.g.* the hydrophobic cluster at the top of TM2 (Leu^195^, Val^198^, and Ala^199^), which is also essential to the function of the CGRP receptor (24). There are also common and common-differential residues situated in ECL2 (Asp^280^, Trp^283^, and Ile^284^); due to the position of the disulfide bond in our AM receptor models, these lie in close proximity to the upper TMs. Indeed, many of these common and common-differential residues are also essential for the activation of the CGRP receptor by both CGRP and AM (26). ECL2 is particularly important in activation in class A and B GPCRs (26, 59, 61).

Cys^282^ in ECL2 forms an essential conserved disulfide bond with Cys^212^ in TM3 in both the AM receptors and in the CGRP receptor (26). However, this bond does not appear to be critical to activation of the AM_2_ receptor (or the CGRP receptor). The smaller pocket in the AM_2_ receptor causes tighter packing of the common and common-differential residue network around the top of the TMs; this more restrained environment may limit the movement of the side chain of either Cys^212^ or Cys^282^ and allow the AM_2_ receptor to tolerate an unpaired cysteine residue without detrimental perturbation of its structural integrity and thus activation of the AM_2_ receptor. Significantly, ECL3 in the CGRP receptor adopts a similar conformation to the AM_2_ receptor (results not shown). In the more open AM_1_ receptor, this C212A or C282A mutation is fatal to receptor activation, but precise verification of the mechanism is beyond the scope of our models. However, we propose that the greater effect of mutation at the common but differential residues in the AM_1_ receptor is related to its degree of openness and hence stability. Thus, we note that other residues, such as Lys^213^, Tyr^277^, and Tyr^278^, that are predicted to stabilize ECL2 also show more pronounced effects on mutation in the AM_1_ receptor despite generally adopting similar interactions (Lys^213^ and Tyr^278^) in both structures, presumably because the mutated AM_1_ receptor structure is less stable than the mutated AM_2_ structure.

These changes, especially those in ECL3, serve to alter the depth, volume, shape, and composition of the model binding pocket. Whereas the overall position of the docked peptide and in particular the Phe^18^ side chain in the peptide binding pocket does not change significantly, the number of close neighbors to the Phe^18^ side chain does. These changes have significant implications for the design of therapeutics that are either specific to the AM_1_ or AM_2_ receptors to treat receptor-specific pathophysiologies or conversely to harness the common effects of both receptors. Druggability screening highlighted two different druggable pockets for small molecules in the AM_1_ and AM_2_ receptors. This indicates scope for specific ligand design by targeting the additional and differential druggable residues of the two pockets, which lie within the TM domains.

The drug scores of 0.81–0.97 and 0.81–0.91 for the AM_1_ and AM_2_ receptors, respectively, are clearly above the 0.5 threshold, indicating that they are druggable. Significantly, both sites display an appropriate balance of hydrophobic and polar residues, as required for a druggable site (62). Moreover, the difference in electrostatic potential for these receptors adds to the rationale for the design of selective AM_1_ or AM_2_ ligands. In addition, the structural model of the AM peptide structure (Fig. 1*E*) is distinctly different from that of glucagon and probably many other class B peptide ligands and so may also be useful in substrate-based drug design, especially because there are differences in the two loop regions. The CRF1R structure shows a narrow drug-bound channel that sits below the level of our peptide binding site. Interestingly, both druggability servers indicate additional druggable sites in this region (20).

We have based our current study on the measurement of cAMP as the canonical signaling pathway for CLR. It is important to note that GPCRs, such as this, also have the capacity to signal through alternative pathways, and it will be important to consider these in future studies (55). It is possible that some residues will have a greater or lesser role, depending on the pathway measured, indicating further conformational differences in the receptors.

In summary, we suggest that the change in the predicted conformation of ECL3 and hence the different TM binding pockets in the AM_1_ and AM_2_ receptors is due to association with different RAMPs, as described above. The existence of distinct peptide and small molecule binding pockets with different properties has implications for the design of selective therapeutics, whether they be small molecules or peptides. This could facilitate the design of ligands to harness the individual physiological roles of the two AM receptors, validating the receptors as drug targets.

Our data support the idea that RAMPs act allosterically to modify the conformation of CLR. This could lead to a range of possible outcomes, including biasing the receptor toward different ligands or signaling pathways. Two recent reports have suggested this mechanism for RAMP effects on the related calcitonin receptor (63, 64). Allostery between protomers in receptor oligomers could be a broad mechanism for generating diversity in GPCR function.

## Author Contributions

H. A. W., J. J. G., M. C., R. S. A., and M. G. conducted experiments. C. A. R., M. P., J. M. W. R. M., A. L., and A. C. contributed to the modeling. P. W. R. H., T.-Y. Y., and M. A. B. were responsible for peptide synthesis. J. B., D. R. P., M. J. W., and A. C. C. contributed receptor mutants to the study. A. A. P. provided data that were used for the modeling. C. A. R., H. A. W., D. R. P., and D. L. H. interpreted the experiments and wrote the paper.

## Supplementary Material

Supplemental Data

## Footnotes

6 The abbreviations used are:

AM: adrenomedullin
CGRP: calcitonin gene-related peptide
CLR: calcitonin-like receptor
ECD: extracellular domain
ECL: extracellular loop
GPCR: G protein-coupled receptor
RAMP: receptor activity-modifying protein
TM: transmembrane domain
Fmoc: *N*-(9-fluorenyl)methoxycarbonyl
RA: relative activity
PTH: parathyroid hormone
PDB: Protein Data Bank
DOPE: discrete optimized protein energy
GCGR: glucagon receptor
CRF1R: corticotropin releasing factor 1 receptor.
