# Supplementary material for: Receptor Activity-modifying Proteins 2 and 3 Generate Adrenomedullin Receptor Subtypes with Distinct Molecular Properties
Source: J Biol Chem. 2016 Mar 24;291(22):11657–75. doi: 10.1074/jbc.M115.688218 (PMC4882435; doi:10.1074/jbc.M115.688218)
Supplement: Supplemental Data [file supp_291_22_11657__index.html]

Receptor Activity-modifying Proteins 2 and 3 Generate Adrenomedullin Receptor Subtypes with Distinct Molecular Properties — RAMP Effects on Adrenomedullin Receptors — Supplemental Data 

# Receptor Activity-modifying Proteins 2 and 3 Generate Adrenomedullin Receptor Subtypes with Distinct Molecular Properties

## Supplemental Data

**Files in this Data Supplement:**

- Supplemental PDB2
- Supplemental PDB1
